# Reduced Myelin Basic Protein and Actin-Related Gene Expression in Visual Cortex in Schizophrenia

**DOI:** 10.1371/journal.pone.0038211

**Published:** 2012-06-01

**Authors:** Paul R. Matthews, Sharon L. Eastwood, Paul J. Harrison

**Affiliations:** Department of Psychiatry, University of Oxford, Oxford, United Kingdom; Università di Napoli Federico II, Italy

## Abstract

Most brain gene expression studies of schizophrenia have been conducted in the frontal cortex or hippocampus. The extent to which alterations occur in other cortical regions is not well established. We investigated primary visual cortex (Brodmann area 17) from the Stanley Neuropathology Consortium collection of tissue from 60 subjects with schizophrenia, bipolar disorder, major depression, or controls. We first carried out a preliminary array screen of pooled RNA, and then used RT-PCR to quantify five mRNAs which the array identified as differentially expressed in schizophrenia (myelin basic protein [MBP], myelin-oligodendrocyte glycoprotein [MOG], β-actin [ACTB], thymosin β-10 [TB10], and superior cervical ganglion-10 [SCG10]). Reduced mRNA levels were confirmed by RT-PCR for MBP, ACTB and TB10. The MBP reduction was limited to transcripts containing exon 2. ACTB and TB10 mRNAs were also decreased in bipolar disorder. None of the transcripts were altered in subjects with major depression. Reduced MBP mRNA in schizophrenia replicates findings in other brain regions and is consistent with oligodendrocyte involvement in the disorder. The decreases in expression of ACTB, and the actin-binding protein gene TB10, suggest changes in cytoskeletal organisation. The findings confirm that the primary visual cortex shows molecular alterations in schizophrenia and extend the evidence for a widespread, rather than focal, cortical pathophysiology.

## Introduction

Most neuropathological findings in schizophrenia have been reported in the hippocampus or the dorsolateral prefrontal cortex [1]–[3]. This concentration both reflects, and has contributed to, the focus upon these regions as being of central pathophysiological importance [4]–[7]. The neuropathological evidence includes a contribution from many individual (e.g. [8]–[10]) and transcriptomic (reviewed in [11], [12]) studies of gene expression which have shown molecular alterations in these regions. However, it is hard to know whether there is a true predilection of pathology for these areas, since other cortical regions have been far less well examined. Such information is germane to the broader question as to whether the cortical pathophysiology of psychosis is regionally localised or is widespread. This in turn bears upon the issue of its likely neurodevelopmental origins.

A good example of an area which might have been considered neuropathologically ‘unaffected’ in psychosis is the occipital cortex, including the primary visual or striate cortex (Brodmann area [BA] 17). Yet morphometric [13], [14] and gene expression [15]–[18] data indicate that some structural and molecular differences may occur therein. Indeed, in a microarray study surveying several cortical regions, more transcripts were altered in schizophrenia in BA17 than in dorsolateral prefrontal cortex [19].

Here, to address this question further, we report a study of gene expression in BA17 of the Stanley Neuropathology Consortium brain series. We used a two-stage approach. First, we pooled mRNA from two batches of five subjects in each diagnostic group and ran them on nylon arrays. We then took the transcripts which met our criteria for differential expression in schizophrenia, and carried out RT-PCR analysis of each mRNA individually in the whole sample. Since this series of brains also includes bipolar disorder and major depression subjects, we also had the opportunity to address the diagnostic specificity of any alterations.

## Methods

### Post-mortem tissues

A block of frozen primary visual cortex (BA17) tissue was provided from the 60 subjects comprising the Stanley Neuropathology Consortium brain series [20] (Table 1). All material was coded by the Stanley Medical Research Institute, and experiments and analyses conducted blind to diagnostic and other information. The brains were collected at the Uniformed Services University of the Health Sciences (USUHS) between 1998 and 2004. The IRB determined that IRB approval was not needed, since the subjects were deceased and work was done on anonymized, numbered specimens. Verbal consent to brain donation was obtained from next-of-kin, by telephone, and was witnessed by two people who signed a form verifying the fact. Subsequently, the next-of-kin was contacted and interviewed to obtain further information about the deceased. The work described in this paper was carried out in accordance with the Declaration of Helsinki and with ethical approval from Oxfordshire National Health Service Research Ethics Committee B (#O02.040).

**Table 1:** Demographics of Stanley Neuropathology Consortium subjects.

	Control (con)	Schizophrenia (scz)^d^	Bipolar disorder (bip)^e^	Major depression (dep)
Subjects	15	15	15	15
Age (years)	48.1 (10.6)	44.5 (13.1)	42.3 (11.7)	46.5 (9.3)
Sex (M, F)	9, 6	9, 6	9, 6	9, 6
Brain pH	6.27 (0.24)	6.18 (0.24)	6.19 (0.23)	6.18 (0.22)
PMI (hours)^a^	23.7 (9.9)	33.7 (14.6)	32.5 (16.1)	27.5 (10.7)
Freezer storage (months)^b^	92.7 (7.5)	101.8 (7.8)	101.9 (5.6)	105.8 (9.7)
Hemisphere (R, L)	7, 8	6, 9	8, 7	6, 9
Suicides	0	4	9	7
Onset of illness (years)	-	23.2 (8.0)	21.5 (8.3)	33.9 (13.3)
Duration of illness (y)	-	21.8 (11.4)	21.4 (9.2)	12.7 (11.1)
Lifetime antipsychotics^c^	0	52267 (62062)	20827 (24016)	0
Antipsychotics ever	0	14	12	0
Antidepressants ever	0	5	8	10
Mood stabilisers ever	0	0	10	2
Fresh brain weight (g)	1501 (164)	1472 (108)	1441 (172)	1458 (147)
Substance abuse history (1–3 scale)^d^	1.13 (13,2,0)	1.67 (8,4,3)	2.00 (4,7,4)	1.53 (10,2,3)
Alcohol use history (1–3 scale)^e^	1.27 (11,4,0)	1.69 (7,3,3)	2.08 (4,3,5)	1.64 (9,1,4)

Mean (SD) where appropriate.

a Differs between groups (ANOVA *P* = 0.147, planned contrasts scz>con *P* = 0.043).

b Differs between groups (ANOVA *P* = 0.003, planned contrasts scz>con *P* = 0.002 and bip>con *P* = 0.002).

c Fluphenazine equivalents (mg), d. 4 paranoid, 1 disorganised, 10 undifferentiated, e. 11 with psychotic features.

d 1 = none; 2 = moderate; 3 = severe. Differs between groups (ANOVA *P = 0.016*, planned contrasts bip>con, *P* = 0.010). The number of subjects in each category is shown in brackets.

e 1 = low; 2 = moderate; 3 = high. Known for 54 subjects. The number of subjects in each category is shown in brackets.

### RNA extraction

RNA was obtained using the single-step method of acid guanidinium thiocyanate-phenol-chloroform extraction [21] with TRI reagent (Sigma, Poole, UK). The concentration of nucleic acids was determined by absorbance spectrophotometry and RNA quality was calculated from the ratio of the optical densities of 28s/18s ribosomal bands on MOPS denaturing gel with ethidium bromide. These values were confirmed with an Agilent Bioanalyzer (Agilent Technologies, South Queensferry, UK) for a subset of 12 samples.

Approximately 10 µg of RNA was treated for 30 mins at 37°C with 1U RQ1 RNase-Free DNase (Promega, Southampton, UK). Because of the sensitivity of array studies to DNA contamination, ribonuclease inhibitors were not used since initial tests suggested that they inhibited the DNase activity. Optimal DNase concentration to minimise RNA degradation but to eliminate DNA contamination were determined in preliminary studies. The solution was made up to 200 µl with nuclease free water and mixed with 200 µl acid (pH 4.5) phenol-chloroform (9∶1) and spun for 5 mins. The top phase was mixed with 200 µl chloroform and spun for 5 mins. The top phase was mixed with 20 µl 4M sodium-acetate and 200 µl isopropanol and left to precipitate overnight at 4°C. The solution was spun for 15 mins at 4°C until an RNA pellet formed. This pellet was washed twice in 75% ethanol, dried for 10 mins, resuspended in nuclease free water, and stored at −70°C.

### Gene Expression Arrays

We used the Atlas Human Neurobiology Array (BD Biosciences Clontech), a double spotted nylon cDNA array with 593 neurobiological genes, several housekeeping genes, and plasmid and bacteriophage DNAs as negative controls.

The arrays were run using RNA pooled from five subjects in each diagnostic group, each subject contributing 2 µg. Pooling was performed due to the cost of the arrays which precluded running individual arrays. Pooling biological samples for arrays has been shown to largely reflect the average of the individual samples, and increases accuracy when few arrays are available to run in each group [22]. We grouped the subjects together for pooling based upon their RNA integrity [23], [24]. We divided each diagnostic group into three pools, reflecting the best, intermediate, and lowest RNA quality (Table 2). Groups to be compared were hybridised to arrays from the same lot.

**Table 2:** Demographic details of the three groups of the subjects pooled for the arrays.

		Control	Schizophrenia	Bipolar	Depression
**A. Best Quality RNA Pools**	RNA Quality (28s/18s)	1.07 (.10)	1.00 (.12)	.97 (.07)	1.08 (.11)
	Age (years)	40.2 (8.8)	46.0 (14.0)	50.6 (9.2)	46.2 (13.1)
	Sex (M, F)	2, 3	4, 1	3, 2	3, 2
	Brain pH	6.32 (.24)	6.10 (.22)	6.30 (.19)	6.24 (.18)
	PMI (hours)	25.8 (11.1)	31.0 (18.7)	21.0 (18.8)	19.2 (10.3)
	Freezer storage (months)	94.8 (9.8)	104.6 (6.8)	100.8 (5.6)	95.6 (8.0)
**B. Medium Quality RNA Pools**	RNA Quality (28s/18s)^a^	.92 (.02)	.82 (.03)	.84 (.03)	.86 (.05)
	Age (years)	54.4 (9.5)	44.4 (14.9)	42.6 (9.3)	47.0 (9.7)
	Sex (M, F)	4, 1	3, 2	2, 3	3, 2
	Brain pH	6.32 (.24)	6.34 (.29)	6.16 (.22)	6.20 (.24)
	PMI (hours)^b^	19.4 (18.6)	36.6 (9.0)	30.6 (7.9)	35.8 (10.7)
	Freezer storage (months)	91.0 (6.0)	100.6 (5.1)	100.2 (17.3)	98.2 (12.3)
**C. Worst Quality RNA Pools**	RNA Quality (28s/18s)	.72 (.10)	.63 (.11)	.52 (.01)	.59 (.11)
	Age (years)	49.6 (10.0)	43.2 (13.3)	33.8 (11.5)	46.4 (6.2)
	Sex (M, F)	3, 2	2, 3	4, 1	3, 2
	Brain pH	6.16 (.26)	6.04 (.18)	6.10 (.28)	6.10 (.22)
	PMI (hours)	26.0 (10.7)	33.4 (17.2)	39.0 (12.4)	27.4 (3.3)
	Freezer storage (months)	92.2 (7.7)	100.2 (11.1)	104.8 (3.4)	93.6 (9.9)

Values are mean (S.D.).

a Differs between groups (ANOVA p = .002; planned contrasts scz<con p<.0005, bip<con p = .002, dep<con p = .010).

b Differs between groups (ANOVA p = .136; planned contrasts scz>con p = .040, dep>con p = .040).

The RNA was reverse transcribed and labelled with ^32^P as per the manufacturer's instructions with minor modifications: in a total reaction volume of 20 µl there was a mixture of 2 µl 5× reaction buffer, 2 µl 10× deoxyribonucleotide triphosphate (dNTP) mix, 3.5 µl of fresh [α-^32^P]dATP (10 µCi/ µl), 1 µl 100 mM DTT, 2 µl Powerscript Moloney murine leukaemia virus enzyme (MMLV), 5.5 µl of 5 or 10 µg of RNA, 2 µl 10× CDS primers. A master mix (buffer, dNTPs, ^32^P, DTT) was prepared at room temperature. The RNA/10× CDS primer mix was heated at 70°C for 2 mins, then 50°C for 2 mins. This was added to the master mix and MMLV and incubated at 50°C for 40 mins, with the reaction ended with 2 µl termination mix.

Probes were diluted to 200 µl total volume with the included buffer NT2 and added to the NucleoSpin Extraction Spin Column, and centrifuged for 1 min. Three times 400 µl of buffer NT3 was added to the column and it was spun for 1 min. 100 µl of buffer NE was then added and the column soaked for 2 mins. The probe was eluted by centrifuging for 1 min. The radioactivity was counted on a liquid scintillation counter. Incorporation of ^32^P was acceptable for the best and medium quality RNA samples but very poor for the worst quality RNA samples, confirming their degradation.

For hybridization, 1 mg of heat denatured sheared salmon testes DNA was added to 10 ml of BD ExpressHyb at 68°C and the array pre-hybridized for 30 mins with continuous agitation at 68°C. C_o_t-1 DNA was added to the probe pool at a concentration of 5% and the mixture incubated for 2 mins at 95–100°C for 2 mins. 5–10 million counts of probe were then added to the pre-hybridization solution and the arrays hybridized for 18 hrs. Arrays were then washed 4×30 mins at 68°C in Wash Solution 1 (2×SSC, 1% SDS) and 1×30 mins in Wash Solution 2 (0.1×SSC, 0.5% SDS), before a final rinse at room temperature in 2× SSC and then water.

Arrays were apposed to X-ray film (Biomax MS; Kodak, USA) with an intensifying screen overnight, for 5 days and for 2 weeks, at −70°C.

### Array Analysis

The array films at different exposures were scanned (16bit greyscale, 200 dpi) on a Microtek ScanMaker 5 with a transparency adapter and analysed using Atlas Image 2.7 (BD Biosciences Clontech). All gene spots were inspected for signal bleed, saturation, marks on the film or membrane, and for intensity differences between the duplicate spots. The intensity threshold for detection was set at twice background for the best quality RNA arrays, and 1.5 times background for the medium quality RNA arrays. The worst quality RNA arrays were not analysed due to very low hybridization signals. Intensities were averaged across the duplicate spots and corrected for background signal.

Two different normalisation strategies were used: normalisation by the global sum of intensities on the array, and by the expression of the three least variable housekeeping genes on the array. Normalisation to the global sum assumes that the overall expression between groups is the same, and is likely to be a fairly conservative approach to detecting differentially expressed genes when enough genes are detected. The least variable housekeeping genes are determined by a stepwise process with the assumption that they will represent a fairly constant proportion of transcripts between samples. The log-ratio of each (non-saturated) housekeeping gene to every other housekeeping gene is taken, and the standard deviation of this value for each gene is taken between the arrays. The mean of the log-ratios with every other gene is the measure of variability for a gene. The gene with the highest value is eliminated from the comparison and the log-ratios between the remaining genes recalculated. This continues in a stepwise fashion until three genes are left. The arithmetic mean of these three genes was used to normalise the array. We implemented both normalization methods using the associated software package Atlas Image 2.7.

The criteria we used to identify transcripts differentially expressed in schizophrenia and to select for subsequent RT-PCR analysis were that the ratio of background corrected signal intensity compared to controls was greater than 1.62 (up or down) for both the best and medium quality pooled mRNA samples and this was in the same direction in both pools. All genes thus identified were re-checked to ensure that the differences were not due to film saturation or other artefacts.

### RT-PCR

Gene expression was measured by semi-quantitative reverse-transcriptase polymerase chain reaction (RT-PCR) [25], [26] with target mRNA levels normalised to the geometric mean of three housekeeping genes (porphobilinogen deaminase, PBGD; glyceraldehyde 3-phosphate dehydrogenase, GAPDH; ribosomal 18S, r18s) amplified in separate reactions on the same reverse-transcribed template [27], [28].

5 µg of RNA per subject was treated with RQ1 RNase-Free DNase (Promega, Southampton, UK) and reverse transcribed with Moloney murine leukaemia virus (mmLV) enzyme (Promega).

All primers were obtained from Eurogentec (Southampton, UK). Optimum conditions, including the linear region of amplification, were determined for each primer. Most primers used three-step PCR reactions consisting of an initial denaturing step (5 mins at 94°C) then a number of cycles (determined for each primer) of three phases: denaturing (94°C for 45 s), annealing (temperature determined for each primer, 1 min unless otherwise indicated) and extension (72°C for 1 min). These cycles were followed by a final extension step (72°C for 3 mins). Reactions using a quick two-step PCR protocol consisted of denaturing (94°C for 1 min) followed by a number of cycles of denaturing (94°C for 30 s) and combined annealing and extension phases (1 min). Primer sequences and the details of amplification are given in Table S1.

PCR reactions were carried out using puReTaq Ready-To-Go PCR Beads (Amersham Biosciences, Little Chalfont, UK). All primer products were confirmed by sequencing, and a subset were also confirmed by restriction enzyme digest. PCR products were fractionated with agarose gels and ethidium bromide and semi-quantitatively measured from the UV optical density of bands using an Alphaimager 3400 gel imager (Genetic Research Instrumentation, Braintree, UK).

All statistical tests were two-tailed and carried out at an uncorrected alpha of 5%. The default analysis was analysis of variance (ANOVA) with planned comparisons of the control subjects versus the three other diagnostic groups. Shapiro-Wilk and Levene's tests (for normality and homogeneity of variance respectively) were carried out on the dependent variables at α = 0.05. Šidák tests were performed for post-hoc contrasts. If normality or homogeneity of variance were violated the variable was log-transformed and re-tested. If the log-transform failed to correct the distribution a Kruskal-Wallis (KW) non-parametric ANOVA was used. For two-sample comparisons, the t-test or Wilcoxon-Mann-Whitney (WMW) test was used as appropriate.

We also inspected for correlations between each normalised mRNA and potential confounding variables (28s/18s ribosomal ratio, age, pH, post mortem interval, and freezer storage time). Any variable showing a significant Pearson (or Spearman where appropriate) correlation at α = 0.05 (and checked visually) was included in an ANCOVA. In secondary analyses, further correlations and contrasts were assessed for other demographic variables including medication history, alcohol and substance use, brain hemisphere, gender, onset and duration of illness, family history of psychiatric illness, and suicide.

## Results

### Results from the pooled arrays

We identified 157 transcripts as being above our threshold for reliable detection on both the high and medium quality pooled RNA arrays. As noted, the low quality arrays were not analysed due to poor ^32^P incorporation and low hybridization signals.

Five genes met our criteria for being differentially expressed in schizophrenia compared to the control subjects. All were decreased in the disorder. Four of the transcripts were significant both by global sum normalization and housekeeping gene normalization (myelin-oligodendrocyte glycoprotein [MOG]); thymosin β-10 [TB10, TMSB10]; β-actin [ACTB]); superior cervical ganglion-10 [SCG10; STMN2]). The fifth, myelin basic protein [MBP] was significantly altered on the high quality array using global sum normalization, and on the medium quality array by housekeeping gene normalization. We then examined these five transcripts in the pooled RNA from subjects with bipolar disorder and with major depression. Relative to the healthy controls, SCG-10 and TB10 mRNAs were decreased in bipolar disorder, and ACTB decreased in major depression. These five transcripts, together with the three reference genes, were taken forward for analysis by RT-PCR.

### Primary analyses of RT-PCR data

**Figure 1:**
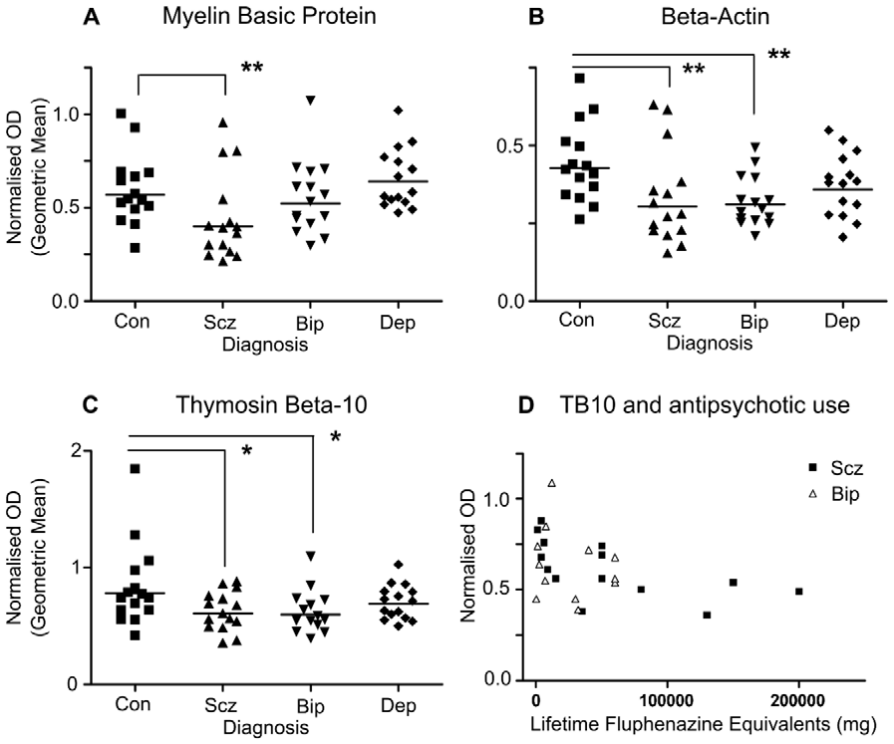
Relative abundance of selected mRNAs in the visual cortex in control subjects and subjects with schizophrenia, bipolar disorder, or major depression. A. Myelin basic protein (MBP) mRNA. B: β-actin (ACTB) mRNA. C: Thymosin β-10 (TB10) mRNA. D: Correlation between TB10 mRNA and antipsychotic drug exposure in patients with schizophrenia (squares) and bipolar disorder (triangles). Values are normalised to the geometric mean of the three housekeeping genes. ** *P*<0.01, * *P*<0.05. See also Table 3.

All sixty subjects (Table 1) were included in the RT-PCRs. The primary analyses for the normalised expression of each target transcript are summarised in Table 3, and positive findings illustrated in Figure 1. None of the reference transcripts, nor the geometric mean thereof, differed between diagnostic groups.

**Table 3:** Relative abundance of mRNAs by RT-PCR, normalised to the geometric mean of the three housekeeping genes.

		Con (*n* = 15)	Scz (*n* = 15)		Bip (*n* = 15)		Dep (*n* = 15)	
Gene	ANOVA *P*-Value	Mean (SD)	Mean (SD)	*P*-Value	Mean (SD)	*P*-Value	Mean (SD)	*P*-Value
MBP	0.004**	0.597 (0.187)	0.444 (0.231)	0.006**	0.553 (0.197)	0.502	0.657 (0.160)	0.368
MBP (ΔE 2)	0.258^1^	0.676 (0.221)	0.619 (0.305)	0.174^2^	0.556 (0.128)	0.079^3^	0.611 (0.148)	0.346^3^
ACTB	0.016*	0.443 (0.125)	0.332 (0.151)	0.005**	0.320 (0.081)	0.008**	0.373 (0.101)	0.137
TB10	0.045*	0.836 (0.352)	0.628 (0.167)	0.018*	0.621 (0.178)	0.014*	0.703 (0.148)	0.230
SCG10	0.703^1^	1.05 (0.212)	1.12 (0.200)	0.461^2^	1.07 (0.248)	0.567^2^	1.09 (0.288)	0.305^2^
MOG	0.881^1^	1.58 (0.162)	1.48 (0.263)	0.249^3^	1.57 (0.296)	0.902^2^	1.53 (0.208)	0.775^2^

*P*-values for individual psychiatric groups are comparisons with the control group.

1 Kruskal-Wallis ANOVA.

2 Wilcoxon-Mann-Whitney test.

3 *t*-test.

** *P*<0.01,

* *P*<0.05.

MBP expression (Table 3; Figure 1A) was reduced in schizophrenia (ANOVA *P* = 0.004; planned contrast schizophrenia<controls, *P* = 0.006). Post hoc testing showed that MBP mRNA in schizophrenia was reduced compared to major depression (Šidák *P* = 0.003) but not to bipolar disorder (Šidák *P* = 0.195). Tkachev at al [29] found that reductions in MBP expression in schizophrenia were specific for transcripts containing exon 2, so we used a second set of MBP primers bridging exons 1 and 3 and found no significant differences in MBP mRNA (all P>0.25). Therefore, the reduction in MBP expression in schizophrenia was specific for transcripts containing exon 2.

ACTB expression (Table 3; Figure 1B) was reduced in schizophrenia (ANOVA *P* = 0.016; planned contrast scz<con *P* = 0.005) and bipolar disorder (planned contrast bip<con *P* = 0.008) compared to controls. Post-hoc tests did not reveal any significant differences between the diagnostic groups (Šidák all *P*>0.1). Merging the bipolar disorder and major depression subjects also shows a significant reduction in the combined mood disorders group compared to controls (ANOVA *P* = 0.012; planned contrast mood<con *P* = 0.017).

TB10 expression (Table 3; Figure 1C) was reduced in schizophrenia (ANOVA *P* = 0.045; planned contrast scz<con *P* = 0.018) and bipolar disorder (planned contrast bip<con *P* = 0.014) compared to controls. Post-hoc tests did not reveal any further differences (Šidák, all *P*>0.1). Combining the bipolar disorder and major depression subjects also shows a reduction compared to controls (ANOVA *P* = 0.042; planned contrast mood<con *P* = 0.035).

MOG and SCG10 mRNAs showed no differences between cases and controls by RT-PCR (Table 3; all *P*>0.1).

### Correlations and secondary analyses

ACTB mRNA was affected by alcohol and substance use. One-way ANOVA revealed a main effect of alcohol use using the three point scale (Table 1; *P* = 0.038), with ACTB mRNA being decreased in the medium (*P* = 0.051) and highest (*P* = 0.018) use groups compared to the lowest use group. Inclusion of alcohol use in the main ANOVA rendered the diagnosis effect a trend (*P* = 0.07) with the planned contrasts remaining significant for scz<con (*P* = 0.012) and as a trend for bip<con (*P* = 0.054). Thus, alcohol use and a diagnosis of schizophrenia were both associated with decreased ACTB mRNA. Classifying subjects by their history of substance abuse revealed a significant reduction in ACTB expression (*P* = 0.028), but including substance abuse in the main ANOVA showed no significant effect due to substance abuse (*P*>0.1) while the reduced ACTB mRNA in schizophrenia remained significant (planned contrast schizophrenia<controls, *P* = 0.031) with a trend for a reduction in bipolar subjects (*P* = 0.095) suggesting that the effect of substance abuse on ACTB mRNA is driven primarily by confounding with psychiatric diagnosis. There were no other significant effects of alcohol use, or of substance misuse, on the mRNAs.

Brain hemisphere, sex, and suicide showed no significant effects, nor interactions with diagnosis, on expression of any of the mRNAs. The normalised mRNAs showed very few correlations with continuous demographic and other variables. The main exceptions were that the ribosomal ratio (a measure of RNA integrity) correlated with normalised SCG10 mRNA (Spearman *r_s_* = 0.319, *P* = 0.013), and MOG mRNA (*r_s_* = 0.325, *P* = 0.011), and age correlated inversely with MOG mRNA (Spearman *r_s_* = −0.312, *P* = 0.015). Inclusion of the respective variable as a covariate did not significantly affect the result of the primary analyses.

With regard to disease-associated variables, in the schizophrenia group, TB10 mRNA correlated negatively with age of onset of illness (*r* = −0.555, *P* = 0.032) and positively with lifetime antipsychotic intake (Figure 1D; *r* = −0.624, *P* = 0.013); the latter also correlated with TB10 mRNA when the bipolar disorder subjects who had taken antipsychotics were included (*r* = −0.429, *P* = 0.029, *n* = 26). No other correlations involving these variables, nor with duration of illness, were observed.

## Discussion

To investigate gene expression in primary visual cortex in schizophrenia, we used an initial array screen of pooled RNA, followed by RT-PCR of transcripts thereby identified as differentially expressed. RT-PCR confirmed reductions of MBP, ACTB and TB10, but not MOG or SCG10, in schizophrenia. ACTB and TB10 were also decreased in bipolar disorder.

MBP is an abundant component of the oligodendrocyte myelin membrane, alternatively spliced from the Golli-MBP gene [30], [31]. MBP is critical for myelin membrane biogenesis and for regulating entry of other proteins into the membrane sheets [32]. Microarray studies of schizophrenia and bipolar disorder have frequently implicated myelin-related genes including MBP, and MOG [11], [12], [29], [33], [34]. Together with evidence for reductions in oligodendrocyte number, these data have been major contributors to the hypothesis that oligodendrocyte dysfunction is important in pathogenesis [35], [36]. However, most prior studies were in frontal cortex, and to our knowledge the present data are the first to suggest that a similar process might occur in occipital cortex. Our study confirmed the specificity of MBP mRNA changes to transcripts containing exon 2 [29]. The basis for this isoform selectivity, and the functionality of exon 2-containing MBP variants are unknown, although it is of interest that this variant is selectively regulated by the Src-protein tyrosine kinase member Fyn [37], a key player in oligodendrocyte maturation and myelination [38].

ACTB (β-actin) serves a structural and motile role in all cell lineages, with actin polymerisation affecting many processes including axonal growth [39], [40], oligodendrocyte myelination [41] and membrane transport [42]. Actin polymerisation is intimately involved in synaptic plasticity and development of dendritic spines [43]–[47] and it is preferentially found there [47], [48], as well as serving a structural role in other parts of the synaptic complex [49] particularly in excitatory synapses [50]. ACTB also has a range of other roles related to the cytoskeleton. Given its pleiotropic nature, reduction of ACTB in schizophrenia may have a range of causes and consequences. For example, expression of other actin-related or cytoskeletal proteins has been implicated in schizophrenia and bipolar disorder [33], [51]–[53], and several schizophrenia susceptibility genes interact with actin and the cytoskeleton, including DISC1 [54], calcineurin [55], Akt1 [56] and dysbindin [57]. Or, the reduction in ACTB mRNA may be a correlate of differences in cellular or dendritic morphology and cytoarchitecture [1]–[3], [13], [14], which may itself be in part genetically influenced [58], [59].

ACTB is often used as a normalizing, or housekeeping, gene. Our finding that it is decreased in visual cortex in schizophrenia and bipolar disorder indicates that caution is required in this respect, notwithstanding negative findings in two prior studies of schizophrenia in this region [18], [60] and the equivocal [23] or negative results [18], [24], [61], [62] in other cortical regions. The effect of alcohol and substance use on ACTB expression suggests that this factor should also be examined if ACTB is to be used as a housekeeping gene in human brain.

Thymosin β-10 is a small polypeptide, implicated primarily in cancer biology, including motility, apoptosis, and angiogenesis, and in inflammation [63], [64]. It is expressed in neurons and in oligodendrocytes [65], [66], and is abundant in fetal brain [67], [68]. Its specific neural functions are unclear, but it is notable that its primary role is to sequester actin, by binding to actin monomers and regulating actin polymerisation [64]. As such, the decreases in TB10 and ACTB may be related, potentially in an antagonistic way. That is, reduced β-actin levels would result in reduced globular-actin (G-actin) and a consequent reduction in the polymerisation of filamentous-actin (F-actin); reduced TB10 would result in less G-actin being sequestered, and consequently promote F-actin formation. Therefore, the reduction in TB10 expression could be compensatory for reductions in β-actin, or vice versa. Further studies are needed to tease apart the functional interplay between ACTB and TB10 to clarify the interpretation of the present findings. Intriguingly, MBP also binds to actin [69] and affects actin polymerization [70]. Hence one may speculate that the reductions in ACTB, TB10 and MBP expression may all reflect a common process, or convergent functional sequelae.

Our study had several limitations. First, the arrays had very limited coverage by current microarray standards (with only 157 transcripts reliably detected) so many transcripts could be altered in schizophrenia and be undetected here, including other myelin or cytoskeletal transcripts. Our finding of five altered transcripts (∼3% of total) is comparable to the proportion affected in contemporary microarray studies of psychosis, Second, we did not confirm whether changes in abundance were seen in the encoded proteins. Third, although we inspected for potential confounding variables – and controlled for them statistically where appropriate – we cannot rule out such factors entirely.

In summary, we confirm that the visual cortex is not ‘spared’ by the molecular neuropathology of schizophrenia, in that the expression of several genes is altered. Our results extend the list of affected transcripts therein to include myelin basic protein and two β-actin-related genes. Overall, the emerging picture is that all areas of neocortex surveyed thus far in schizophrenia show, to at least some extent, evidence of involvement. One may also infer that the neurodevelopmental or other processes which underlie the changes also affect processes common to the neocortex, in addition to others which may be distinctive to specific cortical areas, circuits, or cell populations. Finally, the finding that two of the transcripts were changed similarly in bipolar disorder as in schizophrenia is consistent with the overlapping gene expression profiles between these two disorders seen in other regions [71], [72], and provides the first evidence to our knowledge that the molecular neuropathology of bipolar disorder [73], [74] may likewise involve the occipital cortex.

## Supporting Information

Table S1 **RT-PCR primers.** ^#^Lupberger J, Kreuzer KA, Baskaynak G, Peters UR, le Coutre P, et al. (2002) Quantitative analysis of beta-actin, beta-2-microglobulin and porphobilinogen deaminase mRNA and their comparison as control transcripts for RT-PCR. Mol Cell Probes 16: 25–30. ^b^Gutala RV, Reddy PH (2004) The use of real-time PCR analysis in a gene expression study of Alzheimer's disease post-mortem brains. J Neurosci Methods 132: 101–107. ^c^Verrall L, Walker M, Rawlings N, Benzel I, Kew JN, et al. (2007) d-Amino acid oxidase and serine racemase in human brain: normal distribution and altered expression in schizophrenia. Eur J Neurosci 26: 1657–1669. ^d^Humanised from rat - Hashimoto M, Ino H, Koda M, Murakami M, Yoshinaga K, et al. (2004) Regulation of semaphorin 3A expression in neurons of the rat spinal cord and cerebral cortex after transection injury. Acta Neuropathol (Berl) 107: 250–256. ^e^Quick PCR.
(DOC)
