# Supplementary material for: Reduced Myelin Basic Protein and Actin-Related Gene Expression in Visual Cortex in Schizophrenia
Source: PLoS One. 2012 Jun 1;7(6):e38211. doi: 10.1371/journal.pone.0038211 (PMC3365879; doi:10.1371/journal.pone.0038211)
Supplement: Table S1 — RT-PCR primers. #Lupberger J, Kreuzer KA, Baskaynak G, Peters UR, le Coutre P, et al. (2002) Quantitative analysis of beta-actin, beta-2-microglobulin and porphobilinogen deaminase mRNA and their comparison as control transcripts for RT-PCR. Mol Cell Probes 16: 25–30. bGutala RV, Reddy PH (2004) The use of real-time PCR analysis in a gene expression study of Alzheimer's disease post-mortem brains. J Neurosci Methods 132: 101–107. cVerrall L, Walker M, Rawlings N, Benzel I, Kew JN, et al. (2007) d-Amino acid oxidase and serine racemase in human brain: normal distribution and altered expression in schizophrenia. Eur J Neurosci 26: 1657–1669. dHumanised from rat - Hashimoto M, Ino H, Koda M, Murakami M, Yoshinaga K, et al. (2004) Regulation of semaphorin 3A expression in neurons of the rat spinal cord and cerebral cortex after transection injury. Acta Neuropathol (Berl) 107: 250–256. eQuick PCR. (DOC) [file pone.0038211.s001.doc]

**Table S1. RT-PCR primers**

| **Gene** | **Primers** | **Primer Concentration (ng.μl^-1^)** | **Cycles** | **CDNA (ng)** | **Annealing Temp (°C)** |
| --- | --- | --- | --- | --- | --- |
| PBGD^a^ | as: agatggctccgatgg, s: tgcaacggcggaagaaaac | 5 | 33 | 52 | 60^e^ |
| GAPDH^b^ | as: gaagatggtgatgggatttc, s: gaaggtgaaggtcggagtc | 2.5 | 31 | 26 | 60 |
| Ribosomal 18S^c^ | as: ccatccaatcggtagtagcg, s: gtaacccgttgaaccccatt | 5 | 29 | 26 | 60 |
| β-actin^d^ | as: ctcctgcttgctgatccacat, s: caaagacctgtacgccaacac | 5 | 29 | 26 | 60 |
| MBP | as: taggtaacaggggcaagtgg, s: ctgagcagatttagctgggg | 5 | 29 | 52 | 60 |
| MBP  (ΔE 2) | as: taggtaacaggggcaagtgg, s: ctctggcaaggactcacacc | 5 | 29 | 52 | 60 |
| MOG | as: ttccaggtggaagagtagc, s: ttgatccccactttctgagg | 5 | 40 | 104 | 58 |
| SCG-10 | as: ggtggcttcaagatcagctc, s: gcaatggcctacaaggaaaa | 5 | 30 | 52 | 60 |
| TB10 | as: gtcccttcagagacccacag, s: agctgaagaaaacggagacg | 5 | 28 | 52 | 60 |
